# Immune checkpoint inhibitor-related colitis assessment and prognosis: can IBD scoring point the way?

**DOI:** 10.1038/s41416-020-0882-y

**Published:** 2020-05-18

**Authors:** Vincent Ting Fung Cheung, Tarun Gupta, Anna Olsson-Brown, Sreedhar Subramanian, Sarah Christina Sasson, Jonathan Heseltine, Eve Fryer, Elena Collantes, Joseph J. Sacco, Munir Pirmohamed, Alison Simmons, Paul Klenerman, Mark Tuthill, Andrew S. Protheroe, Meenali Chitnis, Benjamin Peter Fairfax, Miranda Jane Payne, Mark Ross Middleton, Oliver Brain

**Affiliations:** 1 Translational Gastroenterology Unit, John Radcliffe Hospital, University of Oxford, Oxford, OX3 9DU UK; 2 NIHR Oxford Biomedical Research Centre, Oxford University Hospitals NHS Foundation Trust, John Radcliffe Hospital, Oxford, OX3 9DU UK; 3 The Clatterbridge Cancer Centre NHS Foundation Trust, Clatterbridge Road, Birkenhead, Wirral CH63 4JY UK; 4 Institute of Translational Medicine, University of Liverpool, Crown Street, Liverpool, L69 3BX UK; 5 Department of Gastroenterology, Royal Liverpool University Hospital, Royal Liverpool and Broadgreen University Hospitals NHS Trust, Prescot Street, Liverpool, L7 8XP UK; 6 Department of Cellular Pathology, John Radcliffe Hospital, University of Oxford, Oxford, OX3 9DU UK; 7 Oxford Cancer and Haematology Centre, Churchill Hospital, Oxford University Hospitals NHS Foundation Trust, Old Road, Oxford, OX3 7LE UK

**Keywords:** Melanoma, Outcomes research, Cancer immunotherapy, Non-small-cell lung cancer, Toxicology

## Abstract

**Background:**

Immune checkpoint inhibitors (ICI) improve survival but cause immune-related adverse events (irAE). We sought to determine if CTCAE classification, IBD biomarkers/endoscopic/histological scores correlate with irAE colitis outcomes.

**Methods:**

A dual-centre retrospective study was performed on patients receiving ICI for melanoma, NSCLC or urothelial cancer from 2012 to 2018. Demographics, clinical data, endoscopies (reanalysed using Mayo/Ulcerative Colitis Endoscopic Index of Severity (UCEIS) scores), histology (scored with Nancy Index) and treatment outcomes were analysed.

**Results:**

In all, 1074 patients were analysed. Twelve percent (134) developed irAE colitis. Median patient age was 66, 59% were male. CTCAE diarrhoea grade does not correlate with steroid/ infliximab use. G3/4 colitis patients are more likely to need infliximab (*p* < 0.0001) but colitis grade does not correlate with steroid duration. CRP, albumin and haemoglobin do not correlate with severity. The UCEIS (*p* = 0.008) and Mayo (*p* = 0.016) scores correlate with severity/infliximab requirement. Patients with higher Nancy indices (3/4) are more likely to require infliximab (*p* = 0.03).

**Conclusions:**

CTCAE assessment does not accurately reflect colitis severity and our data do not support its use in isolation, as this may negatively impact timely management. Our data support utilising endoscopic scoring for patients with >grade 1 CTCAE disease, and demonstrate the potential prognostic utility of objective histologic scoring.

## Background

Immune checkpoint inhibitors (ICIs) have become standard-of-care treatment for melanoma,^1,2^ and more recently for non-small-cell lung cancer (NSCLC)^3^ and renal cancer.^4^ In metastatic melanoma they have revolutionised care, raising the median life-expectancy from months to years, and leading to long-term remission in a growing proportion of cases.^1^

Immune checkpoint proteins regulate immune activation to limit inflammatory responses. In the presence of persistent antigen, these evolutionarily conserved pathways mediate immune response ‘exhaustion’, reducing the inflammatory stress inflicted on the organism. Checkpoint proteins are expressed on various immune cell subsets, including (but not restricted to) T cells, B cells, and dendritic cells.

Licensed ICIs fall into several categories: anti-cytotoxic T-lymphocyte-associated protein 4 (CTLA-4) (ipilimumab, tremelimumab), anti-programmed cell death protein-1 (PD-1) (nivolumab, pembrolizumab, cemiplimab) and anti-programmed death ligand-1 (PD-L1) (avelumab, atezolizumab, and durvalumab). PD-1 inhibition can be used as monotherapy, in combination with chemotherapy for NSCLC, or in combination with CTLA-4 inhibitors in metastatic melanoma and renal cell carcinoma. Use of ICIs is predicted to increase substantially in the coming years for an expanding range of cancers both as single agents and in combination regimens.^5–7^

As their mode of action is to aid T-cell activation, it is unsurprising that ICIs also have an immune-mediated side-effect profile. These immune-related adverse events (irAEs) most commonly affect epithelia (e.g. skin and gastrointestinal (GI) tract), but also solid organs (resulting in hepatitis,^8^ pancreatitis, and thyroiditis^9^). Inflammation of the GI tract (enterocolitis) is the most frequent cause of significant morbidity and cessation of ICI. It may rarely lead to life-threatening complications such as colonic perforation. Combination therapy with CTLA-4 and PD-1 inhibitors is associated with a significantly higher incidence and severity of irAEs (including colitis) than single-agent PD-1 inhibition.^10^

Oncological guidelines developed from clinical trials’ data recommend using the National Cancer Institute’s Common Terminology Criteria for Adverse Events (CTCAE),^11^ to diagnose and assess severity of diarrhoea and colitis.^12–14^ The GI CTCAE is based predominantly on clinical symptoms (i.e. stool frequency over baseline for assessing diarrhoea; and abdominal pain, blood in stools, peritoneal signs, and life-threatening consequences for assessing colitis). It is unclear whether CTCAE grading correlates with subsequent outcomes and guidance of management decisions. If we can identify patients who are likely to have protracted course at outset, we could then introduce additional immunosuppression earlier in the disease course. The utility of CTCAE has not been tested in terms of its effectiveness in diagnostics and prognostication of immunotherapy-related colitis (irAE colitis).

In idiopathic inflammatory bowel disease (IBD), it has been increasingly recognised that objective markers are required to accurately assess disease severity and aid clinical decision-making. Examples include combined clinical and biochemical scores like Truelove and Witts criteria^15^ for acute severe ulcerative colitis (UC), which correlates with requirement for rescue therapy or colectomy,^16^ and validated endoscopic scoring like the Ulcerative Colitis Endoscopic Index of Severity (UCEIS),^17^ which correlates with UC outcomes.^18^ More recent data in IBD suggest that using histological scores in UC, such as the Nancy index,^19^ can most accurately predict clinical outcomes.^20^ Recent reports suggest endoscopic and histological assessment of irAE colitis may also correlate with disease outcomes.^21,22^

Guidelines advise using corticosteroids as first-line treatment for irAE colitis with anti-TNFα therapy (infliximab) second line.^12–14^ In practice, there is considerable variation in the initiation, dose number and frequency of infliximab administration. For irAE colitis patients with not only steroid-responsive but also steroid-dependent disease, there is no consensus on when or how to escalate therapy.

We have analysed a large cohort of ICI-treated patients to describe the real-world incidence, assessment, and management of irAE colitis in two tertiary referral centres. With regards to irAE colitis, we sought to:

1. describe real-world practice of its management;
2. determine the utility of the CTCAE classification of diarrhoea and of colitis grade as a tool for its assessment and prognosis;
3. determine whether established IBD biochemical markers (C-reactive protein (CRP), haemoglobin or albumin) correlate with disease outcome;
4. determine whether IBD endoscopic scores or histological scores correlate with clinical outcome;
5. describe the treatment outcomes when current guidelines are followed.

## Methods

An observational retrospective cohort study was performed from January 2012 to October 2018 at the Clatterbridge Cancer Centre NHS Foundation Trust (linked with Royal Liverpool University Hospitals NHS Trust) and Oxford University Hospitals NHS Foundation Trust.

### Patient selection

All adult patients who received ICI (ipilimumab, nivolumab, pembrolizumab, ipilimumab + nivolumab (combination)) for metastatic melanoma, NSCLC or renal/urothelial cancer as standard-of-care were included. Using the electronic patient record (EPR) and oncology prescribing database (at Oxford, EPR is Cerner Millennium® and oncology prescriptions are made using Aria®; at Liverpool, Meditech is used for both systems), patient demographics, clinical data, endoscopic and histopathological reports, and treatment outcomes were recorded. All irAE enterocolitis patients had GI symptoms presenting after ICI administration. Patients where an alternative diagnosis was made, e.g. infection or use of non-steroidal anti-inflammatory drugs, were excluded. Monitoring for ICI response was generally performed every 3 months.

### Definition of diarrhoea and irAE colitis

We conducted a comprehensive review of the EPR and oncology prescribing databases to confirm the diagnosis of ICI-related diarrhoea or colitis based on the decision of the treating oncologist or gastroenterologist. Diarrhoea was defined on CTCAE version 5.0 ^11^ on review during data collection using the primary data. irAE colitis was defined as diarrhoea requiring steroid/infliximab therapy for resolution and/or with endoscopic/histological confirmation. Oncology guidelines on managing ICI-related colitis do not currently mandate endoscopic assessment, and therefore patients are mostly initiated on steroid treatment prior to endoscopy.^12–14^ Onset of colitis was defined from start of ICI to date of diarrhoea.

### Severity of colitis

There is no standard way of assessing severity apart from CTCAE, which as a predominantly subjective clinical score is of uncertain value. Patients were, therefore, divided into severity categories depending on the nature and length of treatment required to achieve colitis resolution. A standard weaning steroid course for an IBD flare is 60 days so we used this as one delineator of outcome and severity. Patients were divided into three categories: (1) mild-moderate colitis where diarrhoea settled rapidly following a course of steroids ≤60 days; (2) refractory or moderate-severe colitis requiring steroids >60 days; and (3) severe colitis requiring infliximab ‘rescue therapy’.

### IBD biomarkers

Markers commonly used in UC to assess severity and prognosticate (CRP and haemoglobin in Truelove & Witts score,^15^ or albumin in the Ho index^23^) were analysed to determine if they correlated with severity of colitis and treatment outcome.

### Endoscopic analysis

We analysed the subset of patients for whom endoscopic data were available (*n* = 40, 30%) in a blinded fashion with Joint Advisory Group on gastrointestinal endoscopy (JAG)-accredited endoscopists (V.T.F.C. and O.B.) separately scoring the images taken at endoscopy on UCEIS^17,24^ and Mayo scores^25^ (Supplementary Fig. 1a, b). Where there was disagreement, V.T.F.C. and O.B. assessed the images again and came to a consensus. Both endoscopists and pathologists were blinded during scoring to clinical outcome.

### Histological analysis

We analysed a subset of patients for whom histological slides were available (*n* = 45). Two expert GI pathologists, E.F. and E.C., separately scored the slides on the presence (or absence of) ulceration, acute inflammatory cells infiltrate and chronic inflammatory infiltrate then calculated the Nancy index^19^ (Supplementary Fig. 1c). The histopathologists then assigned an overall histological pattern grading to each patient based on one of the following types:

- focal active colitis—occasional foci of acute inflammation, in the absence of chronic inflammation or significant crypt injury;
- lymphocytic colitis—increase in intraepithelial and lamina propria lymphocytes, in the absence of crypt architectural distortion;
- collagenous colitis—increase in thickness of the subepithelial collagen plate and increased lamina propria lymphocytes, in the absence of crypt architectural distortion;
- IBD-like—active chronic inflammation with goblet cell depletion and crypt architectural distortion;
- Non-steroidal anti-inflammatory drug (NSAID) /infectious-like—predominantly acute, superficial inflammation with attenuation of crypt and/or surface epithelium.

The histological specimens analysed against treatment outcome were all acquired at the index scope when diagnosing irAE colitis, and prior to infliximab use. There was a discrepancy between the number of endoscopies (*n* = 40) and histopathology scored (*n* = 45) because only endoscopies with adequate images were included in the analysis. We also analysed all available paired data (from both index and follow-up sigmoidoscopies) to determine whether endoscopic scoring correlates in general with histological scoring in irAE enterocolitis (*n* = 80).

### Statistical analysis

Continuous data are presented with mean (with range) or median with interquartile range. Non-continuous data are presented as patient numbers and percentages. Differences between groups were determined using the unpaired non-parametric Mann−Whitney, Kruskal−Wallis test or Brown−Forsythe (one-way) ANOVA. Categorical data were compared by the non-parametric Fisher’s exact or chi-squared test. To determine if there is a linear relationship between variables, least squares fit was used. Survival curves were calculated using the Mantel−Cox method. Statistical analysis was performed using Graphpad PRISM^TM^ (Ver 8.1) and IBM SPSS^TM^ (Ver 25) software. A *p* value < 0.05 was considered statistically significant, and where multiple comparisons were performed a Bonferroni correction was made as detailed in individual table legends.

## Results

### Presentation and treatment of irAE colitis

A total of 1074 patients were identified across the two centres (Table 1) with 12% (*n* = 134) of patients developing colitis with a median time of onset 60 days (IQR 28−88 days). There was a predominance of male patients in the cohort (59%), and a median age of 66 (Supplementary Table 1). Patients who developed G4 diarrhoea did so earlier after ICI induction (median onset 33 days) than those who developed milder G1/2 diarrhoea (median onset 60 days). Sixty-three percent of patients were admitted, with the median inpatient stay being 7 days. Time to irAE colitis, hospital stays related to colitis and treatments used are shown in Table 1.

**Table 1:** Clinical features and management outcomes of patients with ICI-related colitis. *p* value in column denotes differences between Group A, Group B and Group C by Kruskal−Wallis test for continuous data or chi-square test for categorical data.

	Group A: Ipilimumab monotherapy (*n* = 189)	Group B: Anti-PD-1 monotherapy (*n* = 728)	Group C: Combination ipilimumab and nivolumab (*n* = 157)	*p* value	Total
Patients with colitis (*n*, %)	42 (22)	41 (6)	51 (32)	2 × 10^−23^	134 (13)
Onset of colitis since start of treatment (median days, interquartile range)	64 (35–91)	69 (29–150)	40 (20–65)^††, ‡^	<0.05	60 (28–88)
Median age of colitis patients (years, interquartile range)	67 (57–76)	70 (58–76)	63 (56–68)	<0.05	66 (57–72)
Male sex in colitis patients (*n*, %)	28 (67)	23 (56)	30 (59)	0.95	81 (60)
Colitis patients with hospital admission	17 (40)	19 (46)	38 (75)	<0.0001	64 (48)
Days to first admission from onset of diarrhoea (Median, interquartile range)	4 (3–6)	3 (0–5)	5 (3–10)^††^	<0.05	4 (3–7)
Time from colitis onset to endoscopy (Median, interquartile range)	12 (4–18)				
Length of first hospital stay in days for patients requiring admission (median, interquartile range)	4 (3–8)	5 (3–8)	6 (3–10)	0.37	5 (3–9)
Treatment					
Any steroids (*n*, %)	38 (90)	29 (71)	50 (98)	<0.001	117 (87)
IV steroids (*n*, %)	12 (29)	14 (34)	38 (75)	<0.001	58 (43)
Days of IV steroids in those receiving (median, interquartile range)	3 (2–4)	5 (3–8)	4 (3–6)	0.16	4 (3–6)
Total days on any steroids (median, interquartile range)	59 (51–92)	60 (31–102)	66 (49–117)	0.53	62 (47–100)
Infliximab (*n*, % total)	4 (10)	8 (20)	17 (33)	<0.01	29 (22)
Colectomy (*n*, % total)	2 (5)	0 (0)	1 (2)	0.35	3 (2)

Note patients who had anti-PD-1 therapy then anti-CTLA-4 therapy sequentially (or vice versa) because of progression on first-line treatment were recorded as monotherapy at the time of colitis.

*n/a* not applicable.

^††^*p*< 0.01 compared with Group B by Mann−Whitney test.

^‡^*p* < 0.03 compared with Group A by Mann−Whitney test with Bonferroni correction.

**Fig. 1:**
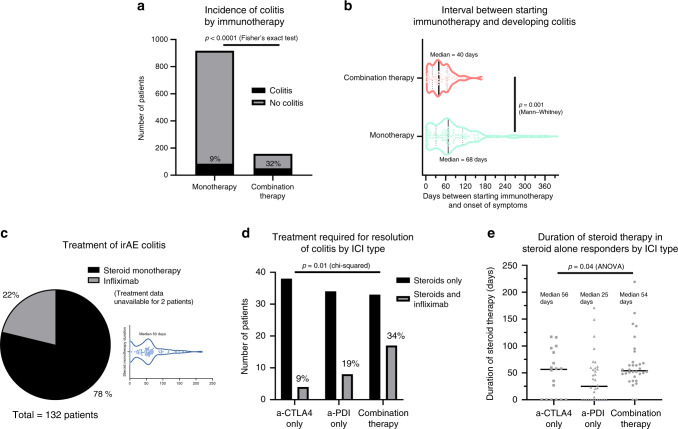
Presentation and treatment of immunotherapy-induced (irAE) colitis in this cohort (*n* = 1074) of patients. **a** The incidence of colitis in single vs. combination immunotherapy (9% vs. 32%; Fisher’s exact test: *p* < 0.0001). **b** Onset of colitis after immunotherapy initiation (median 40 days in combination therapy vs. 68 days in monotherapy; Mann−Whitney test: *p* = 0.001). **c** 22% or 29 patients required infliximab for resolution of their colitis. Median duration of steroids in those who were treated with steroids alone was 50 days. **d** Number of patients requiring steroids monotherapy vs. steroids plus infliximab rescue therapy for treatment of their colitis; subdivided by immunotherapy regimen. Percentages requiring infliximab denoted in figure. Chi-squared test: *p* = 0.005 for difference between the CTLA4 and the combination therapy groups. **e** Mean duration of steroids in patients whose colitis responded to steroid monotherapy alone (patients requiring infliximab excluded); subdivided by immunotherapy regimen (median 56 days in anti-CTLA-4, 25 days in anti-PD-1, 54 days in combination; ANOVA: *p* = 0.04) (N.B. Data unavailable for 50% of patients in the aCTLA4 cohort).

Patients treated with combination therapy had a significantly higher risk of developing colitis vs. those treated with monotherapy (Fig. 1a; 32% vs 9%; *p* < 0.0001). Treatment with single-agent ICI resulted in a later onset of colitis compared to those patients given combination therapy (Fig. 1b). Twenty-two percent of patients received rescue therapy with infliximab either as single or multiple doses (Fig. 1c) at a median of 50 days (Fig. 1c inset), with three undergoing a colectomy (2.3%; two had ipilimumab and one had combination). Median time to giving infliximab was 13.5 days with maximum out to 76 days (Supplementary Fig. 2a). Time to giving infliximab did not correlate with the duration of colitis after receiving the infliximab (i.e. earlier infliximab does not equal shorter colitis for our cohort) (Supplementary Fig. 2b). Patients receiving two more doses of infliximab had no statistically significant difference in duration of diarrhoea (Supplementary Fig. 2c), although this could be confounded, and our study is not designed to answer this specific question. Treatment data were missing for two patients, so the treatment analysis includes 132 patients.

Figure 1d shows the treatment required for resolution of irAE colitis by immunotherapy type with combination therapy patients more likely to require infliximab compared to anti-CTLA-4 or PD-1 monotherapy. For those patients who responded to steroids alone, the duration of steroids required was shorter in those receiving anti-PD-1 monotherapy (median 25 days) compared to either anti-CTLA4 monotherapy (56 days) or combination therapy (54 days) (Fig. 1e). There was no statistical difference in the duration of steroids in those who received infliximab and steroids, and those treated with steroid monotherapy for >60 days (median 92 and 94 days; Table 1). Contrary to a previous report,^26^ our data did not show that giving infliximab earlier led to shorter duration of colitis (Supplementary Fig. 3), although our study was not designed to answer this specific question. In three patients in our cohort, immunosuppression in addition to steroids and infliximab was used and improved the symptoms (vedolizumab in two and mycophenolate mofetil in one; all were melanoma patients who had combination therapy).

Unlike in idiopathic IBD, there was no clear association between smoking status and irAE colitis susceptibility in our cohort (Supplementary Fig. 4). Differences in age and sex were also not associated with incidence of irAE colitis (Supplementary Fig. 4). In a sub-group of 456 patients, those patients with prior IBD (*n* = 8) and those with other autoimmune diseases (*n* = 29) were successfully treated with ICI without flares, and these patients were not at an increased risk of developing irAE colitis (Supplementary Fig. 5). All patients continued prior maintenance IBD therapy throughout the duration of ICI.

A sub-group analysis showed that there was no difference in overall survival between those that developed irAE colitis and those that did not, nor in those that had infliximab and those that did not (Supplementary Fig. 6).

### CTCAE as predictor of disease severity and clinical course

**Fig. 2:**
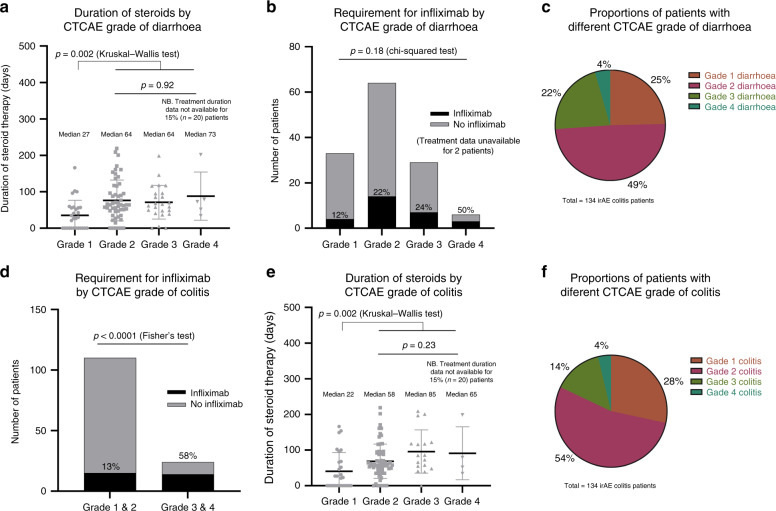
CTCAE as predictor of disease severity and clinical course. **a** Duration of steroids by CTCAE grade of diarrhoea shows difference between G1 and higher (G1 median 27 days, G2 64 days, G3 64 days, G4 73 days) (Kruskal−Wallis G1 vs. rest: *p* = 0.002) but no difference between other grades (Kruskal−Wallis: *p* = 0.92) (N.B. Treatment duration data not available for 15% (20) of patients). **b** Requirement for infliximab by CTCAE grade of diarrhoea shows no difference (chi-squared test: *p* = 0.18). **c** Proportion of patients by CTCAE grade of diarrhoea. **d** Requirement for infliximab by CTCAE grade of colitis shows patients with grade 3/4 colitis were more likely to be treated with infliximab than those with grade 1/2 (Fisher’s exact: *p* < 0.0001). **e** Duration of steroids by CTCAE grade of colitis shows difference between G1 and higher (G1 median 22 days, G2 58 days, G3 85 days, G4 65 days) (Kruskal−Wallis G1 vs. rest: *p* = 0.002) but no difference between other grades (Kruskal−Wallis: *p* = 0.23). **f** Proportion of patients by CTCAE grade of colitis.

CTCAE classification of diarrhoea correlates poorly with duration of steroids and infliximab requirement, which were used as indirect markers of irAE colitis severity. In this analysis the CTCAE was only able to differentiate patients with G1 diarrhoea from the remainder of patients in terms of duration of steroids (Fig. 2a). There was no statistical association between CTCAE grade of diarrhoea and infliximab requirement (Fig. 2b). Seventy percent of our cohort were recorded as having moderate diarrhoea (G2/3), which afforded little discriminatory prognostic value (Fig. 2c). Immunotherapy toxicity guidelines^12–14^ and local treatment protocols advise escalation to infliximab for grade 3/4 colitis if symptoms persist despite corticosteroids beyond 72 h. In line with expectations, these patients were therefore more likely to be treated with infliximab (Fig. 2d). However, as with CTCAE grade of diarrhoea, the CTCAE grade of colitis was unable to prognosticate between grades 2, 3 and 4 of colitis in terms of duration of steroids (Fig. 2e). Figure 2f shows the proportion of patients with each CTCAE grade of colitis and this is similar to the breakdown by CTCAE grade of diarrhoea.

### Blood parameters as predictor of disease severity and clinical course

**Fig. 3:**
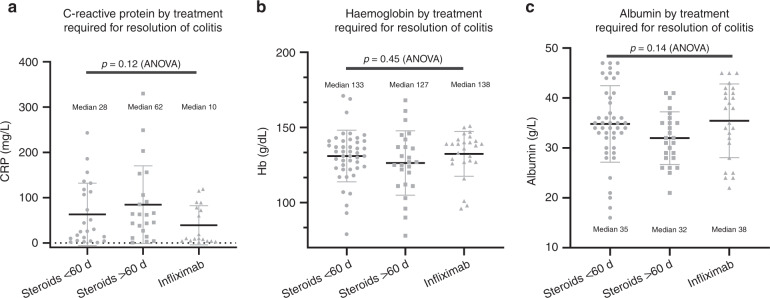
Biochemical markers as predictor of disease severity and clinical course. **a** C-reactive protein by treatment required for resolution of colitis (ANOVA: *p* = 0.12). **b** Haemoglobin by treatment required for resolution of colitis (ANOVA: *p* = 0.45). **c** Albumin by treatment required for resolution of colitis (ANOVA: *p* = 0.14).

CRP, albumin, and haemoglobin do not correlate with severity of irAE colitis (Fig. 3a−c). There were insufficient measures of faecal calprotectin in this dataset to be used as part of this analysis.

### Endoscopic predictors of disease severity and clinical course

**Fig. 4:**
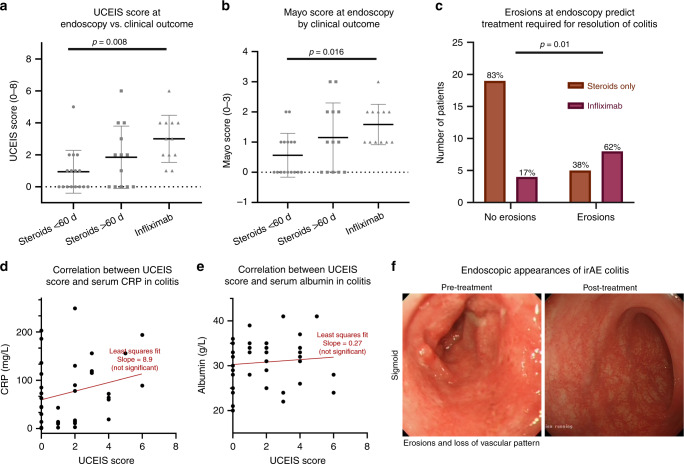
Endoscopic predictors of disease severity and clinical course. Patients classified by the treatment required for the resolution of their colitis into three groups—steroid monotherapy <60 days, steroid monotherapy >60 days, or requirement for infliximab in addition to steroids. UCEIS and Mayo scores as assessed by two independent assessors (*κ* = 0.51, 0.54 respectively). **a** UCEIS score by treatment required for resolution (ANOVA: *p* = 0.008).^30^ **b** Mayo score by treatment required for resolution (ANOVA: *p* = 0.016).^30^ **c** The correlation between the presence of ulcers at endoscopy and the odds of eventually requiring infliximab (Fisher’s exact test: *p* = 0.01, odds ratio 7.6). The correlation between the serum CRP (**d**) (Least squares fit: 8.9; not significant) and serum albumin (**e**) (Least squares fit: 0.27; not significant) with the severity of inflammation as assessed by the UCEIS score. **f** Characteristic endoscopy appearances during irAE colitis and after resolution. UCEIS ulcerative colitis endoscopic index of severity score, CRP C-reactive protein, irAE immunotherapy-related adverse events.

There was moderately good inter-observer correlation for both UCEIS (kappa = 0.51, SE = 0.09) and Mayo scores (kappa = 0.54, SE = 0.09) as assessed by Landis and Koch criteria. We found a reasonable correlation between both UCEIS and Mayo scores and clinical outcome (Fig. 4a, b), and this includes both steroid duration and infliximab use. Erosions were most strongly associated with infliximab requirement (odds ratio 7.0) (Fig. 4c). The discordance between the CRP and albumin and the UCEIS score (Fig. 4d, e) within the subset of patients who underwent sigmoidoscopy further supports the finding that traditional biochemical markers used in idiopathic IBD are of limited use in the assessment of irAE colitis. Figure 4f demonstrates some typical endoscopic findings in irAE colitis.

### Histologic predictors of disease severity and clinical course

**Fig. 5:**
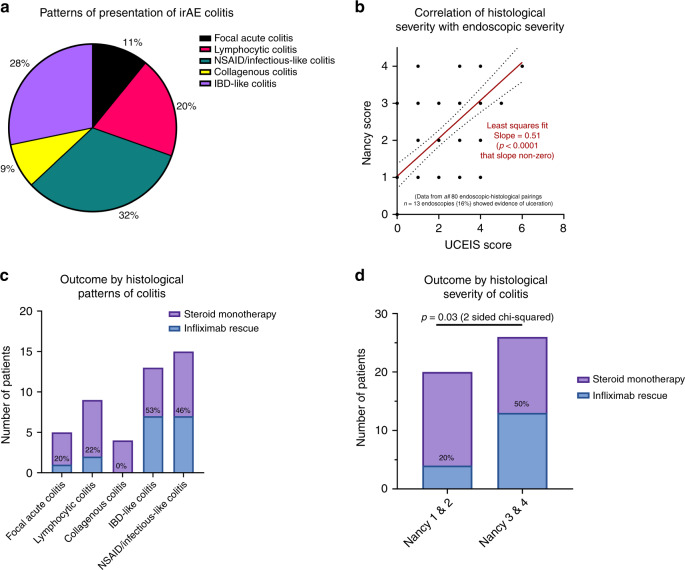
Histopathological predictors of disease severity and clinical course. **a** Patterns of presentation of histology in irAE colitis. **b** Correlation between histological severity and endoscopic severity (Least squares fit = 0.51; *p* < 0.0001). **c** Need for infliximab defined by type of histopathological pattern. **d** Need for infliximab defined by histological severity of colitis (Nancy 1&2 vs. Nancy 3&4; chi-squared test = *p* = 0.03).^30^.

Figure 5a shows the spectrum of histological subtypes seen in irAE colitis. None of the irAE colitis patients had prior NSAID use or had infection, so the NSAID/ infectious-like type histology classification is for those patients who had histological features consistent with a NSAID-related colitis or an infectious colitis. Our data demonstrate a modest correlation between endoscopic severity (as measured by UCEIS score) and histologic severity (as measured by Nancy index; Fig. 5b).

Of the microscopic colitis patients, four had collagenous colitis (one had UCEIS 0, two had UCEIS 1 and one had UCEIS 3) and nine had lymphocytic colitis (two had UCEIS 0, one had UCEIS 1, five had UCEIS 2 and one had UCEIS 4). Therefore, only three patients with microscopic colitis histologic pattern had completely normal endoscopic appearances. There were a further eight patients with UCEIS/ Mayo score of 0 who had mild histological inflammation—two focal active colitis, three IBD-like and three NSAID/ infectious-like.

There is a trend towards those having IBD-like pattern or NSAID/infectious-like pattern being more likely to require infliximab rather than responding to steroids alone (Fig. 5c). None of the patients in our cohort had granulomas. Those patients with a higher Nancy index score (3 and 4) were significantly more likely to require infliximab (Fig. 5d).

## Discussion

We present a retrospective review of 1074 patients given ICI over a 7-year period in two large tertiary centres with 12% developing colitis (consistent with trial data).

Our data suggest that there are no pre-treatment predictive factors that associate with risk of irAE colitis. We confirm that combination ICI is associated with a higher incidence of irAE colitis, earlier presentation and an increased requirement for intravenous steroids and infliximab. We demonstrate for the first time that there is no association between colitis development and smoking status.

The current CTCAE gradings for irAE diarrhoea and colitis, which are heavily reliant on subjective symptom scores, are not accurate enough to be relied on alone as tools for diagnosis and treatment decisions. This has implications for existing clinical guidelines and trial design. Patients with G1 diarrhoea or colitis are more likely to require a shorter duration of steroids, but otherwise CTCAE diarrhoea grades are unable to clearly discriminate steroid responsiveness or infliximab use. CTCAE colitis grades 3/4 do associate with infliximab use, although as current guidelines suggest infliximab treatment at these grades, interpretation of this as an objective scale is challenging. There is no association between steroid duration and CTCAE colitis scores 2, 3 and 4. These data suggest CTCAE will, in a significant proportion of patients, provide an inaccurate guide to clinicians regarding the severity of the colitis and the need for treatment escalation.

Our data suggest that biochemical parameters, conventionally used for assessing outcomes in ulcerative colitis, are of limited use for prognostication in irAE colitis. This apparent lack of utility of well-defined biochemical thresholds may reflect that the ICI patient population is older and more comorbid than a typical IBD patient population. CRP is often raised in cancer and haemoglobin often lowered, further complicating interpretation of these tests. Further work is needed to determine if monitoring the change in CRP is useful during treatment of severe irAE colitis.

In our retrospective analysis, endoscopic assessment of irAE colitis is predictive of treatment outcome. Both UCEIS and Mayo scores correlate with steroid duration and need for infliximab. The presence of erosions at endoscopy increases the odds of requiring infliximab seven-fold, complementing previous reports.^27,28^

We have demonstrated for the first time that an objective UC histological score (Nancy Index) correlates with the clinical course of irAE colitis. Importantly the histology score is not purely a surrogate for endoscopic severity, as the correlation between the two is modest. Moreover, whilst the endoscopic scoring is open to the criticism of circular reasoning (a more severe macroscopic appearance may prompt clinicians to use infliximab), the histologic scoring was performed in blinded fashion after colitis resolution. The relationship between objective endoscopic and histologic scores and colitis outcome will need prospective testing, but our data suggest this will provide additional more accurate and objective information to clinicians. This is attractive given the current (and typically sole) use of CTCAE as the assessment and monitoring tool, as its objective performance is patchy at best.

The fact that patients treated with infliximab and steroids have an equally long course of steroids as those treated with steroid alone suggests that infliximab therapy, when used along current conventional guidelines, is only modestly effective. This may partly reflect the inadequacy of basing treatment decisions on CTCAE grade alone. Although some patients respond rapidly, there were a substantial number of patients in whom infliximab does not appear to provide a steroid-sparing effect. A recent publication presented data that earlier use of infliximab might be beneficial for colitis resolution^26^ and another demonstrated vedolizumab may be used in steroid-dependent or refractory cases.^29^ Whilst we agree this makes intuitive sense, we are unable to confirm with our data the association between earlier instigation of infliximab and more rapid colitis resolution (Supplementary Fig. 2b). This may be owing to the limited sample size and the fact this is real-world data from two centres with heterogeneous practice. Only 42% of patients were able to wean steroids within 60 days without recurrence in the steroid monotherapy group. The remaining patients required either a prolonged course of steroids (21%), and/or additional therapy (infliximab ± vedolizumab ± mycophenolate mofetil) (22%). For the remainder of patients (15%), the duration of steroid monotherapy could not be ascertained. A substantial proportion of patients are, therefore, at risk of steroid side-effects from prolonged courses. Maximising infliximab efficacy, aiming for both rapid symptom resolution and reduced steroid use, may be increased by earlier decision making and administering a full induction course (three doses). At present there is no clear consensus on how to enhance care of these patients, but earlier and more accurate clinical decision-making is likely to be improved using objective easy-to-use scores like UCEIS and Nancy Index.

The strengths of our study are the size of the real-world dual-centre cohort, the fact that it includes data from inpatients and outpatients, both CTLA-4 and PD-1 inhibitors, and patients with various cancer types. The data have been collected from high-volume tertiary oncology and IBD centres that manage referrals from regional and national levels.

We recognise the limitations of the study, including that this is a retrospective dataset and that, even between two centres, considerable heterogeneity will exist in both the clinical assessment and management of this condition. In a retrospective dataset there is a risk of circular reasoning regarding endoscopic severity and treatment choice i.e. seeing ulcers may influence the clinician to assess the disease more severe. However, the histologic scoring was not determined until after colitis resolution.

Given the indications for ICIs are increasing rapidly, the incidence of GI irAE will continue to rise. A greater understanding of baseline risk factors, disease biomarkers and prognostic factors is much needed.

We demonstrate that the currently recommended CTCAE grading is inadequate as an assessment tool in terms of diagnostics and prognostication. Our data suggest that objective endoscopic and histological scores are the most accurate methods of ascertaining disease severity and need for rescue therapy. We would advocate early flexible sigmoidoscopy, using endoscopic and histological scores, for those with suspected irAE colitis, particularly for any patient with CTCAE > G1 disease. Given our data, and previous publications indicating the relevance of endoscopic assessment, we suggest that clinical management guidelines are changed to incorporate new algorithms for the detection, investigation and management of irAE colitis. Their development and validation will require larger prospective cohorts.

## Supplementary information

Supplementary File

## Data Availability

The authors confirm that the data supporting the findings of this study are available within the article and its supplementary materials. Supplementary information is available at the *British Journal of Cancer*’s website.
