# Supplementary material for: Immune checkpoint inhibitor-related colitis assessment and prognosis: can IBD scoring point the way?
Source: Br J Cancer. 2020 May 18;123(2):207–15. doi: 10.1038/s41416-020-0882-y (PMC7374736; doi:10.1038/s41416-020-0882-y)

Supplementary Information Text Summary

1. Supplementary Figure and Table Legends (word document)
2. Supplementary Table 1 (word document)
3. Supplementary Figure 1 (word document)
4. Supplementary Figure 2 (word document)
5. Supplementary Figure 3 (word document)
6. Supplementary Figure 4 (word document)
7. Supplementary Figure 5 (word document)
8. Supplementary Figure 6 (word document)

**Supplementary Table 1. Demographics of patients who had ICI with the indications for treatment.** p denotes differences between Group A, Group B and Group C by Kruskal-Wallis Test for continuous data or Chi-Square test for categorical data. **††**p<0.01 compared with Group A by Mann-Whitney test. ‡‡‡‡p<0.00001 compared with Group C by Mann-Whitney test. n/a=not applicable

**Supplementary Figure 1. Endoscopic and Histological Scoring of Inflammatory Bowel Disease (IBD).** S1(a)Ulcerative Colitis Endoscopic Index of Severity (UCEIS) for endoscopic scoring. S1(b) Nancy Index for histological scoring of inflammatory bowel disease.

**Supplementary Figure 2. Duration of steroid exposure and duration of colitis in relation to infliximab.** S2(a) Duration of steroids prior to infliximab. S2(b) Correlation between time of receiving infliximab and duration of colitis post-infliximab. S2(c) Duration of diarrhoea between receiving one dose versus two or more doses of infliximab.

**Supplementary Figure 3. Plot of time taken for diarrhoea to respond to infliximab versus the interval of time in days that elapsed between onset of diarrhoea and giving infliximab demonstrating no correlation, and therefore no benefit to earlier infliximab (Least squares fit: p=0.11; not significant).**

**Supplementary Figure 4. Effect of smoking, age and sex on the risk of colitis in different treatment and cancer categories.** S4(a) The risk of colitis by smoking status in PD-1 monotherapy (Chi-squared test: p=0.80). S4(b) The risk of colitis by smoking status in combination immunotherapy (Chi-squared test: p=0.33). S4(c) The risk of colitis by gender (Fisher’s exact test: p=0.56). S3(d) The risk of colitis by age at time of immunotherapy (t-test with Welch’s correction).

**Supplementary Figure 5. Risk of irAE with prior autoimmune disease and risk of irAE colitis with prior IBD.** S5(a) Risk of any irAE depending on whether patient has prior autoimmune disease or not. S5(b) Risk of irAE colitis depending on whether patient has previous diagnosis of IBD or not.

**Supplementary Figure 6. Survival curves for patients who got irAE colitis or not between the 3 treatment groups and in those receiving infliximab or not.** S6(a) Survival of melanoma patients treated with combination therapy who got colitis and did not (Mantel-Cox test: p=0.53). S6(b) Survival of melanoma patients treated with anti-CTLA-4 therapy who got colitis and did not (Mantel-Cox test: p=0.30). S6(c) Survival of all patients treated with anti-PD-1 therapy who got colitis and did not (Mantel-Cox test: p=0.32). S6(d) Survival in those receiving infliximab or not for their colitis (Mantel-Cox test: p=0.40).

**Supplementary Table 1**

|  | **Group A: Ipilimumab monotherapy** | **Group B: Anti-PD-1 monotherapy** | **Group C: Combination ipilimumab and nivolumab** | **P value** | **Total** |
| --- | --- | --- | --- | --- | --- |
| **Number of patients** | 189 | 728 | 157 | n/a | 1074 |
| **Age (median years, interquartile range)** | 65 (55-72) | 68(60-74)^††‡‡‡‡^ | 64(54-70) | <0.00001 | 66(58-74) |
| **Male sex (%)** | 111 (59) | 430(59) | 90(57) | 0.922 | 631(59) |
| **ICI indication (number, %):**  *Melanoma*  *Renal cell carcinoma*  *Lung-non-small cell*  *Urothelial*  *Other* | 189(100)  0(0)  0(0)  0(0)  0(0) | 242(33)  93(13)  348(48)  35(5)  10(1) | 153(98)  0(0)  4(2)  0(0)  0(0) | n/a | 584(54)  93(9)  352(33)  35(3)  10(1) |

**Supplementary Table 1. Demographics of patients who had ICI with the indications for treatment.** p denotes differences between Group A, Group B and Group C by Kruskal-Wallis Test for continuous data or Chi-Square test for categorical data. **††**p<0.01 compared with Group A by Mann-Whitney test. ‡‡‡‡p<0.00001 compared with Group C by Mann-Whitney test. n/a=not applicable

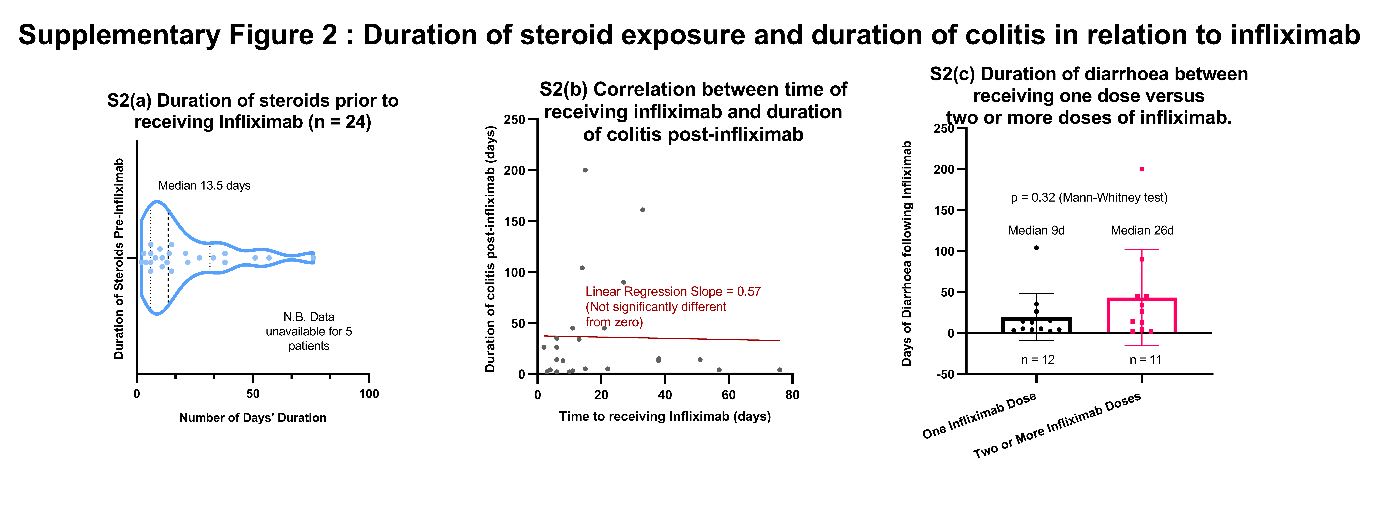


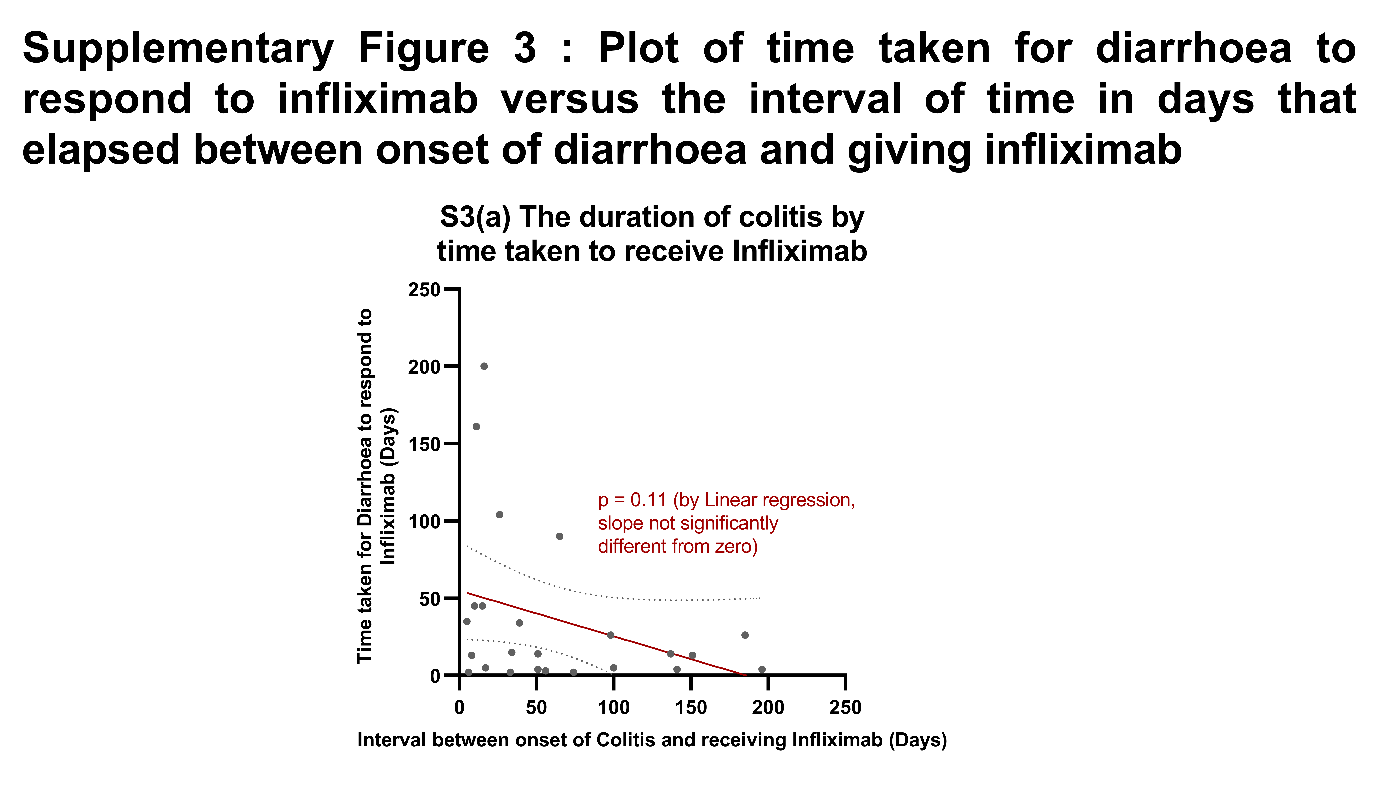


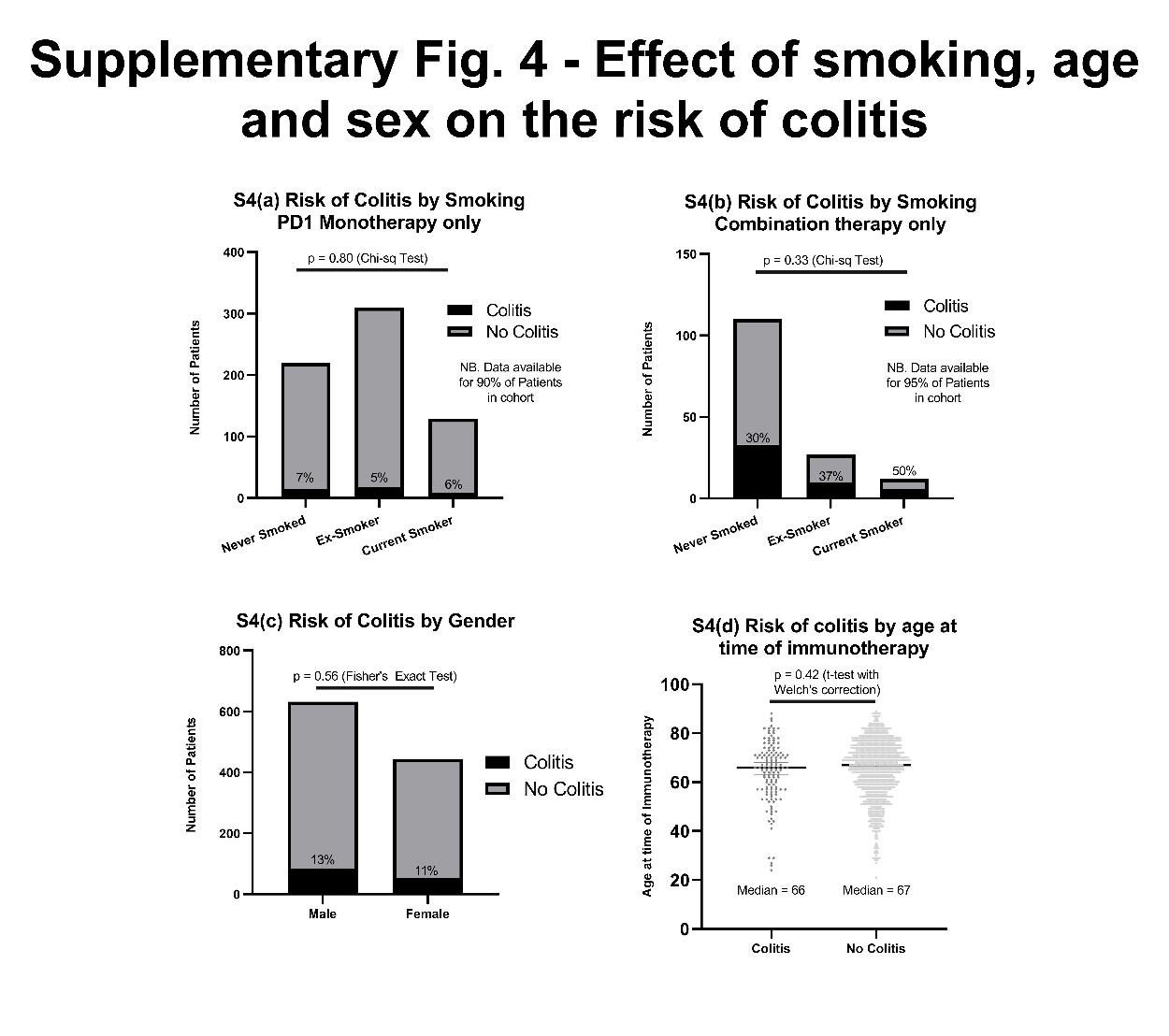


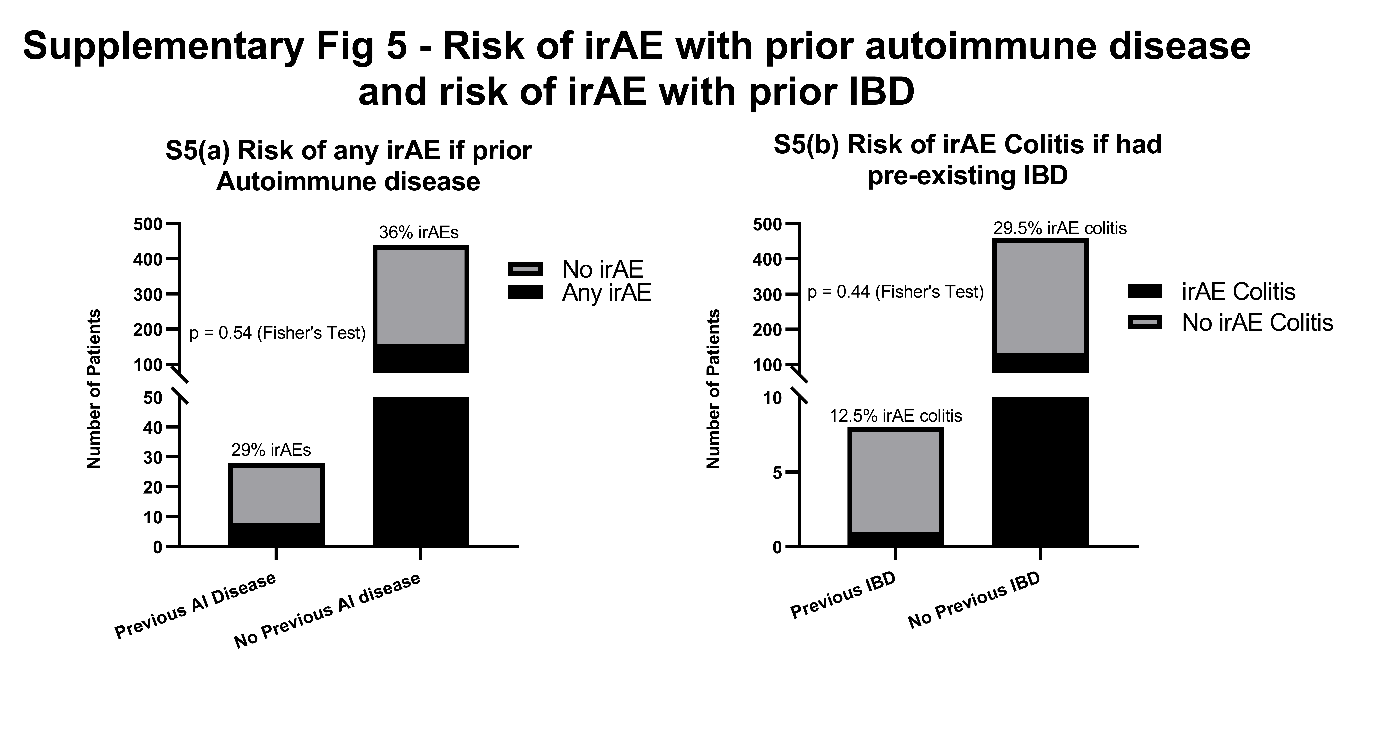


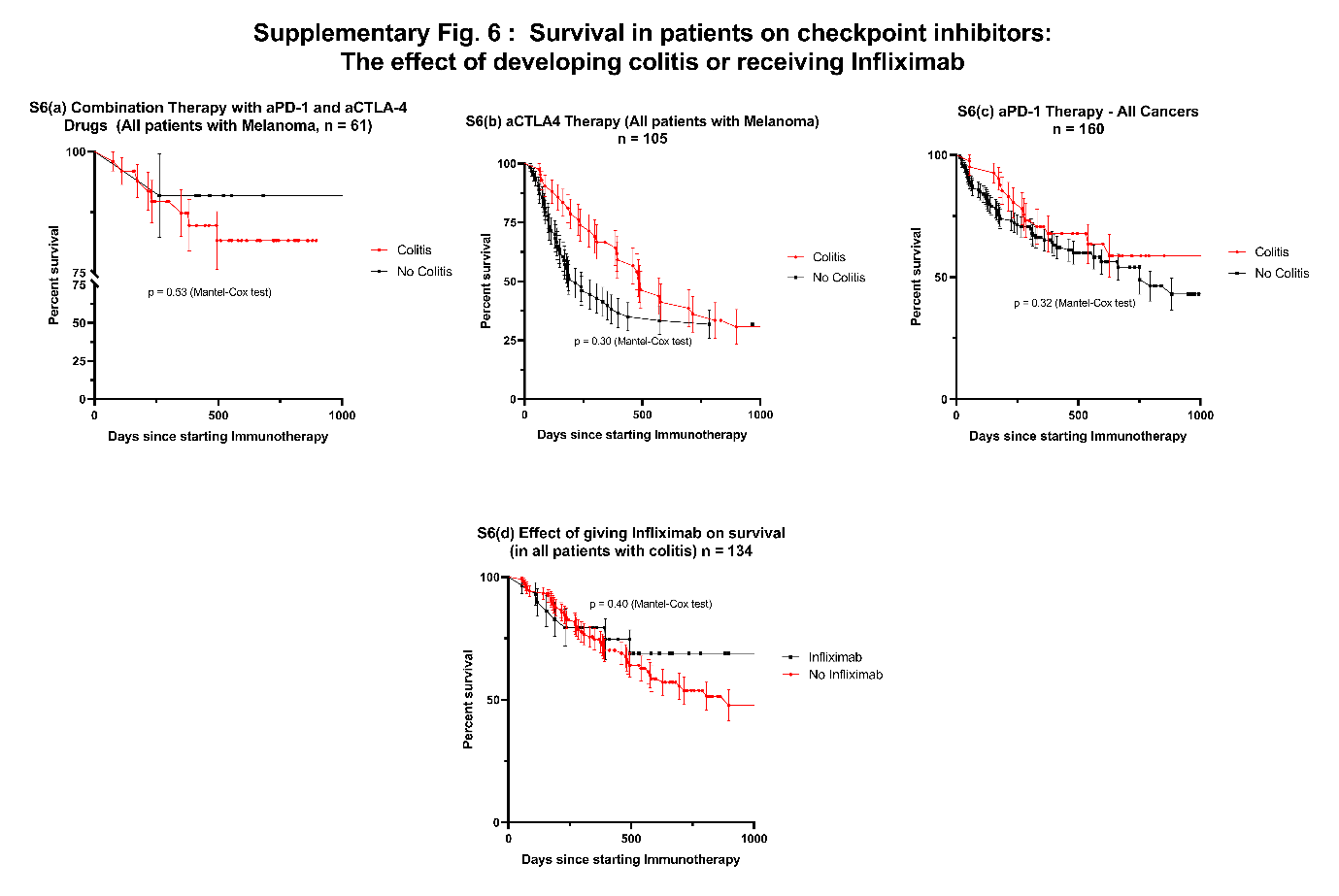

Supplement: Supplementary file 1 — Supplementary File [file 41416_2020_882_MOESM1_ESM.docx]
